# Elderly Learners and Massive Open Online Courses: A Review

**DOI:** 10.2196/ijmr.4937

**Published:** 2016-01-07

**Authors:** Tharindu Rekha Liyanagunawardena, Shirley Ann Williams

**Affiliations:** ^1^ University College of Estate Management Reading United Kingdom; ^2^ School of Systems Engineering University of Reading Reading United Kingdom

**Keywords:** massive open online courses, loneliness, older adults, elderly, eLearning, education, continuing education, computer-assisted instruction

## Abstract

**Background:**

Massive open online courses (MOOCs) have become commonplace in the e-learning landscape. Thousands of elderly learners are participating in courses offered by various institutions on a multitude of platforms in many different languages. However, there is very little research into understanding elderly learners in MOOCs.

**Objective:**

We aim to show that a considerable proportion of elderly learners are participating in MOOCs and that there is a lack of research in this area. We hope this assertion of the wide gap in research on elderly learners in MOOCs will pave the way for more research in this area.

**Methods:**

Pre-course survey data for 10 University of Reading courses on the FutureLearn platform were analyzed to show the level of participation of elderly learners in MOOCs. Two MOOC aggregator sites (Class Central and MOOC List) were consulted to gather data on MOOC offerings that include topics relating to aging. In parallel, a selected set of MOOC platform catalogues, along with a recently published review on health and medicine-related MOOCs, were searched to find courses relating to aging. A systematic literature search was then employed to identify research articles on elderly learners in MOOCs.

**Results:**

The 10 courses reviewed had a considerable proportion of elderly learners participating in them. For the over-66 age group, this varied from 0.5% (on the course “Managing people”) to 16.3% (on the course “Our changing climate”), while for the over-56 age group it ranged from 3.0% (on “A beginners guide to writing in English”) to 39.5% (on “Heart health”). Only six MOOCs were found to include topics related to aging: three were on the Coursera platform, two on the FutureLearn platform, and one on the Open2Study platform. Just three scholarly articles relating to MOOCs and elderly learners were retrieved from the literature search.

**Conclusions:**

This review presents evidence to suggest that elderly learners are already participating in MOOCs. Despite this, there has been very little research into their engagement with MOOCs. Similarly, there has been little research into exploiting the scope of MOOCs for delivering topics that would be of interest to elderly learners. We believe there is potential to use MOOCs as a way of tackling the issue of loneliness among older adults by engaging them as either resource personnel or learners.

##  Introduction

According to a United Nations Report [1], over the last 50 years the number of older people in the world has tripled, and this number will increase exponentially in the next 50 years. In 1950, the number of people aged 60 or over in the world was estimated to be 205 million, with only three countries (China, India, and the United States) having more than 10 million older people. In 2000, the number was 606 million and 12 countries had more than 10 million people aged 60 or over. What is striking is that the rate of increase in the number of people aged 60 or over (1.9%) is significantly higher than that of the total population growth (1.2%). According to the projections, the difference between the two rates is expected to increase, and from 2025-2030 the over-60 age group will be growing 3.5 times faster than the total population.

### Population Aging

Every country is encountering population aging, but each country is at a different stage of transition [2]. Developed countries in general have encountered population aging earlier than other parts of the world and currently almost 20% of the population in developed countries are 60 years old or above, as opposed to developing regions where just 8% of the population is aged 60 or above. In particular, the population aged 60 or over in Europe is projected to be around 37% by 2050, up from 20% in 2000 [1]. However, by 2050, 80% of the world’s older people are likely to be living in low- and middle-income countries [2].

Population aging presents various challenges to society such as an increasing demand for health services, an increasing need for long-term care and social services, and increasing strain on pension and social security systems. Conversely, an aging population will also make important contributions to society as family members, caregivers, volunteers, and being part of the workforce. Fostering good health in older age is a primary factor in preventing isolation and maintaining the independence and productivity of older people [2].

The decline of both mental and physical capacities is a feature of aging, often coupled with the loss of friends and family. For many people, this results in loneliness [3]. Loneliness is a concept that has been defined in a multitude of ways. Cambridge Dictionaries Online [4] interprets it as the “state of being lonely” and further describes “lonely” as being “unhappy because you are not with other people,” while Oxford Online dictionary [5] defines loneliness as “sadness because one has no friends or company.” Victor et al [6] showed that in various studies loneliness has been defined as “perceived deprivation of social contact, the lack of people available or willing to share social and emotional experiences, a state where an individual has the potential to interact with others but is not doing so and a discrepancy between the actual and desired interaction with others” (p. 408).

### Aging and Isolation

In general, older people are at risk of social isolation because of diminished contact with colleagues (possibly due to retirement) and with family and relatives, especially if they are in poor health, disabled or bereaved, or because of their geographic location. According to the charity Age UK [7], there are over 1 million older people in the United Kingdom who feel lonely [8]. The increasingly complicated and busy lifestyles that characterize the contemporary world mean that finding time to visit or spend time with an elderly relative or friend may get deferred unintentionally, leaving elderly people feeling lonely and isolated. Maintaining ties with others is an important aspect of successful aging, which encompasses “the avoidance of disease and disability, the maintenance of high physical and cognitive function, and sustained engagement in social and productive activities,” p. 433 [9].

Research into Internet use and loneliness in older adults has shown promise for the use of online communication to tackle loneliness. For example, a study of 222 Australians who were over 55 years of age showed that the use of the Internet as a communication tool was associated with lower levels of social loneliness [10]. Similar findings are reported by other studies looking at computer and Internet use by older adults [11-13]. Another review [14] found that computers were most commonly used by older adults for the purposes of communication and social support, increased contact with family and friends, especially grandchildren, and dealing with geographic barriers or limited mobility, all of which help tackle loneliness. The same review also reported an increase in attention by researchers to examine the computer use by older adults.

Notess and Lorenzen-Huber [15] discuss the opportunities that e-learning offers to older adults. They identify the added benefits of online learning for older adults who are either geographically isolated or have mobility issues. They acknowledge that the potential of online learning for older adults “is far from realized.” Online communities such as the thirdAGE [16] and SeniorNet [17] are some of the sites that have been offering learning opportunities for older adults; for example, SeniorNet offers courses on literature and poetry [15]. Githens [18] categorizes e-learning programs for older adults as programs for personal growth and social change, workforce development, and workplace learning. The new wave of Massive Open Online Courses (MOOCs) offers courses that address all three of these areas.

### Massive Open Online Courses

Massive open online courses or MOOCs are a recent, but immensely popular addition to the online learning landscape. They offer lectures, forums, quizzes, assignments, and various other learning materials that in general can all be accessed online. Since their emergence in 2008, there have been many commercial and non-commercial platforms dedicated to offering MOOCs, and hundreds of universities have partnered with these platforms to offer courses. The courses are free to register and participate in, thus attracting thousands of participants. The recent offering from the British Council, “Understanding IELTS: Techniques for English language test,” had over 380,000 learners registered on it [19]. With such massive numbers registering on courses, MOOCs present a plethora of challenges and opportunities that are discussed elsewhere in the literature [20,21].

### Elderly Learners in Massive Open Online Courses

According to the latest edX [22] report, 10% of participants (ie, people who register for a course and have actually accessed the course material) on the edX platform are over 50 years old while 4% are over 60 years old [23]. Given that edX has over 1.03 million unique participants, the figure for participants aged over 50 is about 130,000, which is a considerable number. A total of 17% of the participants in the first 21 courses of the FutureLearn platform [24] were 56-65 years old and another 9% were over 66 years old. Thus, the percentage of over-56 age group on the FutureLearn platform is 26% [25]. Given that FutureLearn has over 1.2 million learners (as of March 2015), the number of students over 56 years is likely to be considerable. Looking at the profile of actively engaged MOOC participants in the University of Reading’s course “Begin programming: Build your first mobile game,” where authors are among the educator team, and which has completed five iterations since October 2013, older adult participants reported that they were spending many hours a week on the course.

In this paper, we present demographic data from 10 courses offered by the University of Reading in various disciplines from programming to heart health on the FutureLearn platform, to show that a considerable proportion of elderly learners are participating in these free online courses. We also show that there is currently a lack of scholarly literature investigating this group of learners and their engagement with MOOCs, despite the existence of a few MOOCs that explore aging and related issues. We then argue that the engagement of elderly learners in MOOCs could be used as a way to tackle the social isolation felt by the elderly and that more research in this area should be commissioned to explore whether MOOCs could be used more widely for this purpose.

## Methods

Data for this study were collected using three independent data sources: pre-course survey data for MOOCs, course details that offered subject matter relating to aging, and literature on MOOCs and elderly learners. Next we describe how the data were collected from these sources.

### Pre-Course Survey

We analyzed pre-course survey data for 10 University of Reading courses offered on the FutureLearn platform to identify the proportion of elderly learners engaging in MOOCs in various disciplines. There is no easy way of identifying learners’ demographic data for FutureLearn courses, despite knowing the number of learners registered in a given course. In the pre-course survey sent to all learners at the start of a FutureLearn course, there is a question that captures their age. The question “What is your age group?” is presented as a multiple choice question with the responses: 18 years old or under, 18-25 years old, 26-35 years old, 36-45 years old, 46-55 years old, 56-65 years old, and 66 years old or over. As a FutureLearn partner, the University of Reading receives anonymized data for pre-course surveys, and we have gathered these pre-course survey data for 10 course runs (some courses have more than one iteration considered) for analysis.

We analyzed pre-course survey data for the following courses:

- Obesity: Causes and consequences (Obesity) – two iterations
- Our changing climate: Past, present and future (Climate)
- Our hungry planet: Agriculture, people and food security (Hungry Planet)
- Managing people: Engaging your workforce (Managing People)
- Heart health: A beginner’s guide to cardiovascular diseases (Heart)
- A beginner’s guide to writing in English for university study (English) – two iterations
- Begin programming: Build your first mobile game (Programming) – two iterations

### Courses on Aging

In identifying relevant MOOCs that included topics related to aging, a range of methods was used to obtain related information that would form a more complete dataset for the analysis similar to the method used by Liyanagunawardena and Williams [26].

- The two popular MOOC aggregator sites Class-Central [27] and MOOC-List [28] were searched on March 24, 2015, to identify courses that explored aging and related issues. The search terms “age,” “old,” and “elderly” were used.
- Course catalogues from the MOOC platforms FutureLearn [24], Coursera [29], edX [22], and Canvas [30] were checked to identify MOOCs on aging or related areas (March 24, 2015).
- The list of health-related and medicine-related MOOCs published in Liyanagunawardena and Williams [26] was also consulted to identify relevant courses.

### The Literature

When conducting literature searches, researchers typically use different methods to identify papers to be considered [31,32]. In this study, a literature search was performed using the search term (MOOC* AND ((age* OR elderly) OR old*)) on two large bibliographic databases: Scopus and the Web of Science. We also searched Google Scholar using the same search terms to identify articles that may not be present in the databases. In our searches, the search period was limited to the period from the year in which the first MOOC was run (2008) to the present (2015).These searches were carried out on March 15, 2015.

### Analysis

#### Pre-Course Survey

The response rate and the number of responses received for each course in the pre-course survey are shown in Table 1. As can be seen, there is very good response rate when taken as a percentage of the actual number of “learners” in the course.

**Table 1:** Pre-course survey response numbers.

Course name	Course start date	Pre-course survey responses (N)	N as a percentage of learners^a^
Obesity: Causes and consequences – Obesity 1	June 9, 2014	1073	24.1
Obesity: Causes and consequences – Obesity 2	Feb. 16, 2015	1590	47.0
Our Changing Climate: Past, present and future – Climate	Nov. 10, 2014	1544	33.9
Our Hungry Planet: Agriculture, people and food security – Hungry Planet	Feb. 9, 2015	1931	59.5
Managing People: Engaging your workforce – Managing People	Jan. 12, 2015	3143	26.7
Heart Health: A beginner’s guide to cardiovascular diseases – Heart	Sept. 8, 2014	904	19.3
A Beginner’s Guide to Writing in English for University Study – English 1	Jan. 19, 2015	4973	28.3
A Beginner’s Guide to Writing in English for University Study – English 2	Feb. 17, 2014	1356	10.2
Begin Programming: Build your first mobile game – Programming 1	Oct. 28, 2013	3607	79.8
Begin Programming: Build your first mobile game – Programming 2	Feb. 24, 2014	2657	13.6

^a^In the FutureLearn statistics, “learners are joiners who viewed at least one step in the course” [25].

#### Courses on Aging

Searching Class-Central and MOOC-List aggregator sites with the words “age,” “old,” and “elderly” resulted in a large number of entries. For example, searching with “age” as a keyword resulted in the return of 98 entries in Class-Central and 638 entries in MOOC-List. However, the actual relevant numbers shown in the Results section were very few. Many search results related to course topics such as “age of globalization” or “the age of sustainable development.” The first author analyzed the list of search results manually to identify relevant courses. Only three related courses were found using Class-Central and MOOC-List searches. These were offered on Coursera (two) and FutureLearn (one) platforms.

By browsing course catalogues on selected platforms, another course on the FutureLearn platform was identified. Consulting the recent publication by Liyanagunawardena and Williams [26], two more courses were identified: one on the Open2Study [33] platform and the other on the Coursera platform.

#### The Literature

**Figure 1:**
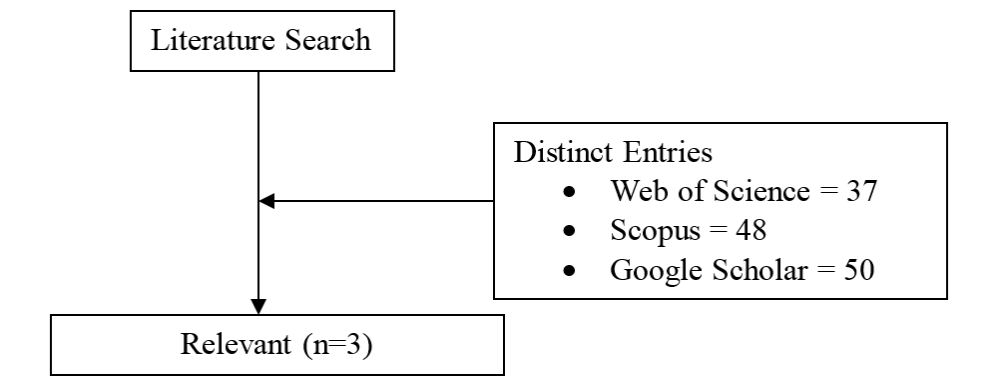
Summary of the literature search.

The database Web of Science returned 38 entries (37 distinct entries) while Scopus returned 49 entries (48 distinct) (March 15, 2015). Reading through the abstracts, only two relevant papers were extracted from the Web of Science entries, and the Scopus entries returned the same two. One additional entry was added to the list by analyzing Google Scholar search results. Many of the returned entries included words such as “information age,” “digital age,” “Internet age,” “computer age,” “age of MOOCs,” “moocher,” “old debates,” “… year old,” “old news,” “external agents,” “intelligent agents,” and “software agents,” while a few included chemical compounds such as MoOC14, MoOC15, and mooceroftii that satisfied the search terms. One paper referred to Mooca (a district in Sao Paulo), while another was about a classical scholar named Moocheomdang Lee Euiyoon. A summary of the literature search is presented in Table 2 and Figure 1.

**Table 2:** The literature search summary.

Source	Entries returned	Distinct entries	Relevant	Non-relevant
Web of Science	38	37	2	36
Scopus	49	48	2	47
Google Scholar	Over 5000	First 50 distinct entries analyzed	1	49

## Results

### Pre-Course Survey

**Figure 2:**
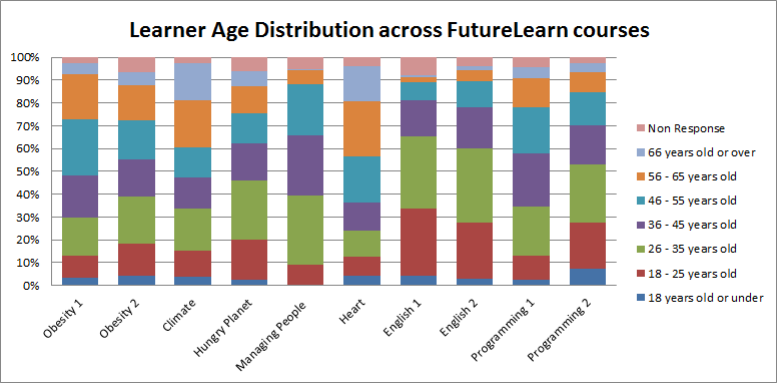
Learner age distribution in University of Reading offerings on FutureLearn.

Using the pre-course survey data for the FutureLearn courses offered by the University of Reading described above, we identified learner demographics. The results are presented in Figure 2.

**Figure 3:**
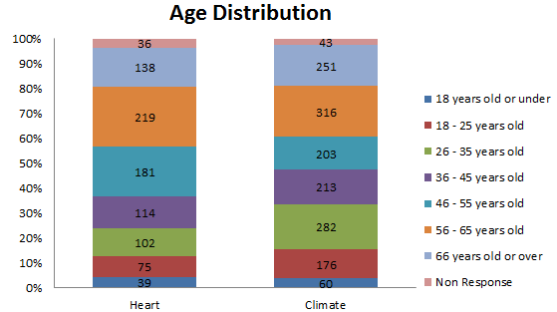
Age distribution of learners.

As Figure 2 shows, a large percentage of elderly learners were observed to have responded to the pre-course surveys in the “Heart health” course and the “Our changing climate” course. Further analysis showed that the “Our changing climate” course had 16.3% (251) learners over the age of 66, while in the “Heart health” course the over-66 age group represented 15.3% (138) of the cohort. In both these courses, the over-66 age group represented the third largest age group in the course (Figure 3).

Observing the over-56 age group in these courses, it can be seen that in the “Heart Health” course nearly 40% of the participants were in this age category (Table 3). It can also be seen that “A beginner’s guide to writing in English for university study” was the least popular course among this age group, followed by the “Managing people” course.

**Table 3:** Percentage of students over 66 and over 56 years old in courses.

Course name	Over-66, % replies	Over-56, % replies
Obesity: Causes and consequences – Obesity 1	4.9	24.9
Obesity: Causes and consequences – Obesity 2	5.7	21.1
Our changing climate: Past, present and future – Climate	16.3	36.7
Our hungry planet: Agriculture, people and food security – Hungry Planet	6.5	18.2
Managing people: Engaging your workforce – Managing People	0.5	6.7
Heart health: A beginner’s guide to cardiovascular diseases – Heart	15.3	39.5
A beginner’s guide to writing in English for university study – English 1	0.6	3.0
A beginner’s guide to writing in English for university study – English 2	1.5	6.7
Begin programming: Build your first mobile game – Programming 1	5.0	17.7
Begin programming: Build your first mobile game – Programming 2	3.8	12.6

### Courses on Aging

Six MOOCs related to aging were found from the searches and are shown in Table 4. Coursera offered three courses, FutureLearn offered two, and Open2Study offered one course. The details of these courses are presented in Table 5.

**Table 4:** Courses relating to aging.

Course name	Class-Central	MOOC-List	Authors’ previous work	Platform course catalogue
Growing old around the globe	Yes	Yes		Yes
Rethinking aging: Are we prepared to live longer?	Yes	Yes		Yes
Aging well: Falls		Yes		Yes
Why do we age? The molecular mechanisms of aging				Yes
Understanding dementia			Yes	
Care of elders with Alzheimer’s disease and other major neurocognitive disorders			Yes	

**Table 5:** MOOCs related to aging.

Course name	Platform	Offered by
Growing old around the globe	Coursera	University of Pennsylvania
Rethinking aging: Are we prepared to live longer?	Coursera	University of Melbourne
Aging well: Falls	FutureLearn	Newcastle University
Why do we age? The molecular mechanisms of aging	FutureLearn	University of Groningen
Understanding dementia	Open2Study	University of Tasmania
Care of elders with Alzheimer’s disease and other major neurocognitive disorders	Coursera	Johns Hopkins University

The Johns Hopkins University now offers “Living with dementia: Impact on individuals, caregivers, communities and societies” course and this too is a 5-week course similar to “Care of elders with Alzheimer’s disease and other major neurocognitive disorders.” Both courses were offered by the University’s School of Nursing and because of this we believe these two to be the same course with an updated course title.

### The Literature

The three articles found to be relevant from the literature search are King et al [34], Sanchez-Gordon et al [35], and King et al [36].

##  Discussion

### Elderly Online Learners

From the above analysis, we have shown that a considerable proportion of elderly learners are already engaging in MOOCs. For example, in the “Heart health: A beginner’s guide to cardiovascular diseases” course, 15.3% of the learners were over 66 years old while another 24.2% were in the 56-65 age group. That is, in this course, 39.5% of the learners were over 56 years old. Observing the spread of elderly learners in the courses offered by the University of Reading, it can be seen that some types of courses are more popular with this age group. Nevertheless, elderly learners do engage in courses in a multitude of disciplines.

However, the available information is insufficient to gauge the geographical spread of these elderly learners. Thus, it is possible that most of these learners are from developed countries with high levels of education, similar to the general MOOC learner demographics shown by other studies. For example, four out of five participants in University of Pennsylvania courses on Coursera platform had a Bachelor’s degree or higher [37], while the data obtained from the first 21 courses of the FutureLearn platform showed that 78% of its participants had a university degree or higher [25].

### Promoting Courses

Elderly learners are likely to have more time to devote to learning. While there are elderly learners who do take part in these free courses, it is possible that there are many others who are not aware that such courses exist. Thus, promoting free online courses to this age group would allow elderly people to become leisure learners. Courses could be promoted at local events (eg, coffee mornings), through charities working with elderly (such as AgeUK), hospitals, libraries, in residential or retirement homes, higher and further education collages, and religious places (such as churches or temples). Providing this information will allow these time-rich elderly learners the opportunity to explore a wide variety of topics of interest via free online courses. Engaging in MOOCs can provide a virtual support group as the learning community helps individuals in their learning and could be a way of instilling a sense of “belonging” to a community and combating isolation. However, as we have shown above there is a lack of research about elderly learners in MOOCs. Research into elderly learners’ engagement in MOOCs and the effects on their well-being would be a worthwhile avenue to explore.

### Creating Courses for Elderly Learners

Elderly learners may have complex accessibility needs. For example, background music in a lecture may work as a stimulus for younger learners but for older people who are more prone to be hard of hearing, it may become an additional barrier to accessing content. Sanchez-Gordon and Luján-Mora [38] show the need to address the Web accessibility needs of elderly learners in MOOCs. They analyze a sample of five Coursera courses for Web accessibility and in two of the three test cases they used, all courses failed to comply with Web accessibility guidelines. Even though these results are based on a small sample, they highlight the need to adhere to Web accessibility guidelines when designing and presenting MOOCs.

It is likely that certain topics will be of special interest to elderly learners. For example, in our data analysis we have shown that the courses “Our changing climate” and “Heart health” had a large proportion of elderly learners in them. Similarly, it is plausible to expect that topics exploring issues such as health problems that are more common in later life, specific interests (eg, travel, history, nature, poetry, baking, or gardening), or historical events of interest to elderly learners will have higher numbers of leisure learners registering on them. On the other hand, courses relating to management of workforce (“Managing people”) and learning English for academic writing (“A beginner’s guide to writing in English for university study”) were less popular among this age group.

Thus, an opportunity exists to engage elderly learners by offering courses with topics that are closer to their interests. Organizations such as AgeUK or the University of the Third Age [39], a movement that provides learning opportunities for retired and semi-retired people and that is run by community members who are typically older adults, could offer or could partner with other institutions interested in offering MOOCs in topics that elderly learners have a special interest in. Courses especially targeted at elderly learners will allow them to study with other people with similar interests, providing a virtual network of connections and friendships.

### Elderly as Resource Personnel

Course providers could use the expertise/experience of elderly learners in other ways; for example, giving elderly learners the opportunity to co-create community courses by providing an open space for discussions and collaborations (eg, see [40]). For example, elderly learners who lived through World War II, the apartheid period in South Africa, or the Spanish Civil War will have their own personal experiences of these events that may differ from “accepted” documentation. Courses exploring these topics and social histories (eg, changes in the kitchen) could be created by building on learners’ personal stories and could become valuable resources for researchers and future generations.

Additionally, highly educated and retired personnel (eg, professors) who are authorities of their field of knowledge, may have spare time and be prepared to share their knowledge for the “greater good.” Therefore, there is an opportunity to utilize this expertise in MOOCs either as content creators, educators, or mentors.

When elderly learners engage with MOOCs, they may be able to spend many hours exploring course materials and related readings. This may be because they are time-rich compared to other learners who may have to continue their studies alongside employment and caring duties. This has certainly been our experience as educators in the “Begin programming” course. Once a course finishes, if the elderly learners have been engaged and successfully completed the course, they may be willing to join subsequent sessions of the same course as mentors. As MOOCs are free courses, the support for participants mainly comes from within the course community. Time-rich elderly participants with life experience are likely to be resourceful mentors. Being able to support others in their learning provides self-satisfaction, and this could be mapped to higher levels of needs (esteem and self-actualization needs) in accordance with Maslow’s hierarchy of needs [41].

### Elderly Learners as Consumers

Organizations wishing to offer MOOCs especially for the elderly participants could seek sponsorship from organizations providing services that are mostly required by older adults: for example, suppliers of stair lifts, mobility scooters, or cruise holidays. This would provide the necessary funding for courses to be created for older adults while the sponsoring organizations would benefit from promotion of their services or products.

### Limitations

In this review, we collected data using various sources. However, due to resource limitations the authors had to limit the search scope. Collecting course details through aggregator sites could have the disadvantage of not including all MOOCs that are on offer as Liyanagunawardena and Williams [26] have shown; however, the authors have consulted other sources to minimize the possibility of such occurrence. Liyanagunawardena et al [21] discuss the limitations of literature searches and the difficulty of including blogs in such analysis. In this study, similar to the study by Liyanagunawardena et al [21], we have discounted blog posts, which could mean that some articles may have been missed.

Not all learners respond to pre-course surveys, and some of those who do take part in the surveys do not want to reveal their age. It could also be argued that time-rich elderly learners are more likely to answer pre-course surveys, thus further skewing results. As the pre-course survey is a self-administered questionnaire, it is not possible to validate the answers provided by respondents. These limitations should be considered when interpreting the results of pre-course survey data.

Another consideration is that the authors looked only at publications and courses presented in English and again because of resource limitations. If there were articles or courses in languages other than English, it would not have been possible to consider them here.

### Conclusions

All over the world, countries are encountering population aging as a result of both increased life expectancy and declining birth rates. Population aging presents both various challenges and opportunities to society. Challenges include increasing demand for health services, long-term care and social services, and increasing strain on pension and social security systems; conversely, older adults can make significant contributions as family members, caregivers, volunteers, and members of workforces. Older people are at risk of social isolation due to a variety of reasons: diminished contacts with colleagues, bereavement, mobility issues, and ill health. The use of the Internet has shown promising prospects for solutions to tackle loneliness in older adults.

In this paper, we have shown the lack of research into the use of Massive Open Online Courses (MOOCs) by elderly learners while at the same time establishing their presence in MOOCs by analyzing MOOC demographic data from 10 courses offered by the University of Reading.

Despite the considerable number of elderly learners participating in these courses, there is lack of data to identify more precisely what other characteristics are shared by these learners. We show that promoting courses to the elderly and creating courses specifically targeting this age group could be another way of tackling loneliness felt by a growing number of older people. We further show that engaging elderly learners as resource personnel in creating and offering MOOCs would help them keep engaged while bringing greater good to society by using the vast knowledge and experience accumulated by older adults.

## Abbreviations

MOOCS: massive open online courses
